# EndMT: New findings on the origin of myofibroblasts in endometrial fibrosis of intrauterine adhesions

**DOI:** 10.1186/s12958-022-00887-5

**Published:** 2022-01-07

**Authors:** Chengcheng Xu, Meng Bao, Xiaorong Fan, Jin Huang, Changhong Zhu, Wei Xia

**Affiliations:** Institute of Reproductive Health, Tongji Medical College, Huazhong University of Science and Technology, 430030 Wuhan, China

**Keywords:** Intrauterine adhesion, EndMT, Fibrosis

## Abstract

**Background:**

Intrauterine adhesion (IUA) is one of the leading causes of infertility and the main clinical challenge is the high recurrence rate. The key to solving this dilemma lies in elucidating the mechanisms of endometrial fibrosis. The aim of our team is to study the mechanism underlying intrauterine adhesion fibrosis and the origin of fibroblasts in the repair of endometrial fibrosis.

**Methods:**

Our experimental study involving an animal model of intrauterine adhesion and detection of fibrosis-related molecules. The levels of molecular factors related to the endothelial-to-mesenchymal transition (EndMT) were examined in a rat model of intrauterine adhesion using immunofluorescence, immunohistochemistry, qPCR and Western blot analyses. Main outcome measures are levels of the endothelial marker CD31 and the mesenchymal markers alpha-smooth muscle actin (α-SMA) and vimentin.

**Results:**

Immunofluorescence co-localization of CD31 and a-SMA showed that 14 days after moulding, double positive cells for CD31 and a-SMA could be clearly observed in the endometrium. Decreased CD31 levels and increased α-SMA and vimentin levels indicate that EndMT is involved in intrauterine adhesion fibrosis.

**Conclusions:**

Endothelial cells promote the emergence of fibroblasts via the EndMT during the endometrial fibrosis of intrauterine adhesions.

## Introduction

Intrauterine adhesion, also known as Asherman’s syndrome, is a condition caused mainly by endometrial damage via an unknown mechanism [1]. Surgical trauma and infection are the two main risk factors for IUA development [2]. Endometrial polyps and chronic endometritis lead to changes in the endometrial microenvironment, which is closely associated with the development of IUA [3–5]. Although IUA is a common and prevalent clinical condition, clinical therapy can only restore the shape of the uterus but is not ideal for restoring physiological uterine function [6]. It has now been shown that intrauterine transfusion of platelet-rich plasma can regenerate endometrial tissue and restore endometrial function [7]. According to a research consensus on the pathogenesis of IUA, persistent inflammation, abnormal oestrogen receptor-oestrogen binding, abnormal endometrial stem cell numbers and functions, endometrial fibrosis and scar formation can result in insufficient self-repair of the damaged endometrium [8–12]. Various molecular factors have been associated with intrauterine adhesions, including transforming growth factor beta (TGF-β), Smad, platelet-derived growth factor, insulin-like growth factor I, matrix metalloproteinases, tumour necrosis factor alpha, vascular endothelial growth factor, interleukin and nuclear factor kappa B subunit 1 [9, 10, 13–18]. To date, mechanistic studies have focused on endometrial fibrosis and demonstrated the involvement of multiple signalling pathways, of which the Hippo, TGF-β, WNT and Sonic Hedgehog signalling pathways are the most closely related to this process [19–21]. The epithelial-to-mesenchymal transition (EMT), an important source of fibroblasts, is also considered a key mechanism underlying endometrial fibrosis [22]. However, research on the mechanisms of IUA fibrosis has not yet progressed to the development of effective clinical treatments, suggesting that other key mechanisms of fibrosis are awaiting discovery.

Fibrosis occurs due to the excessive deposition of extracellular matrix components by fibroblasts recruited to the site of interest [23]. The origin of these fibroblasts is unknown, and no specific anti-fibrotic therapy is available [24]. The endothelial-to-mesenchymal transition (EndMT) is a process by which endothelial cells lose some of the characteristics of differentiation and acquire features more characteristic of mesenchymal cells. Involvement of the EndMT has been identified in inflammation, fibrosis, cardiac development, haemodynamic abnormalities and tumour formation [25]. In fibrotic diseases, activated myofibroblasts induce the EndMT process via the increased production of fibrotic molecules, eventually leading to fibrosis in organs such as the heart, kidneys, lungs, skin and eyes [25, 26]. The conversion of endothelial cells into fibroblasts during the EndMT was originally described in the context of experimental wound repair [27]. Endothelial cells have also been reported to contribute to fibroblast production and aggregation in fibrotic diseases of the lung, heart, eye, kidney and other organs [28–31]. The EndMT is characterised by the loss of intercellular junctions between endothelial cells, which acquire an invasive and migratory phenotype and exhibit upregulated expression of mesenchymal cell markers such as alpha-smooth muscle actin (α-SMA), fibroblast-specific protein-1 and vimentin. These changes are accompanied by the downregulation of endothelial cell-specific markers, including platelet and endothelial cell adhesion molecule 1 (CD31) and vascular endothelial calcium adhesion protein [25]. The EndMT has been observed in endometriosis, a disease of endometrial fibrosis [22, 32], suggesting that the EndMT is also highly likely to occur during IUA formation.

Therefore, we hypothesise that endothelial cells are converted into mesenchymal cells via the EndMT during the progression of intrauterine adhesion disease. These mesenchymal cells promote the production of fibrotic molecules, leading to endometrial fibrosis.

## Materials and Methods

### Animal models

The study was approved by the Institutional Animal Care and Use Committee of Tongji Medical College, Huazhong University of Science and Technology. Adult female Sprague–Dawley (SD) rats (8 weeks of age) were purchased from Spelford Biotechnology Co. (Beijing, China). The experiments were performed after a 1–2-week period of adaptive feeding, during which each rat was provided with chow and fresh water under the same control conditions (21 ± 3 °C, 12-h/12-h light/dark cycle, lights on at 8:00 a.m.). The experimental study involving three groups: the IUA group, sham-operated group and blank control group. For animals in the IUA and sham groups, the right uterine horn was subjected to double damage or sham surgery (IUA-R or Sham-R, respectively) during the estrous phase, and the left uterine horn was used as a control (IUA-L and Sham-L, respectively). The rats were anaesthetised with sodium pentobarbital and placed in the supine position. The abdomen was shaved and disinfected three times with iodophor, and a transverse incision of approximately 3 cm was made on the lower abdomen. The procedure was performed with reference to the method described by Li et al. [33, 34]. In brief, IUA was modelled by mechanical and infectious injury. To create a mechanical injury, the endometrium was scratched with a spatula to abrade the surface of the uterine cavity. To create an infection injury, bacterial lipopolysaccharide (LPS) surgical cotton thread was left in the uterine cavity; the end of the surgical thread was left in the abdomen, and the abdominal incision was sutured. After 48 h, the tail wire was gently pulled, and the LPS surgical cotton thread was removed from the uterus. In the sham-operated group, the surgical incision was made and sutured without infection injury. This method of modelling produces a more clinically appropriate representation of IUA. The animals were euthanised in batches on days 3, 7, 14 and 28.

### HE staining

The uterus was fixed in 4% paraformaldehyde for 24 h and then embedded in paraffin. Paraffin-embedded tissue sections were cut, dewaxed and hydrated, and stained using an HE dye set (G1003, Wuhan Servicebio Technology Co., Ltd., China). The stained sections were observed under a light microscope (Olympus, Japan). The number of glands was counted and IUA was evaluated in images of the sections.

### Masson staining

Paraffin-embedded tissue sections were dewaxed, hydrated, and stained using a Masson dye kit (G1006, Wuhan Servicebio Technology Co., Ltd.). The sections were observed under an optical microscope (Olympus) to identify blue-stained collagen fibres, and the area of labelled collagen fibres relative to the overall field of view was calculated using ImageJ software (National Institutes of Health, USA).

### Immunofluorescence

Immunofluorescence staining with antibodies specific for α-SMA (ab7817, Abcam; dilution, 1:1,000), vimentin (A19607, ABclonal, dilution, 1:1,00), CD31 (ab182981, Abcam; dilution, 1:1,000) and snail (A11794, ABclonal, dilution, 1:1,00) was performed. In brief, paraffin-embedded tissue sections were dewaxed and hydrated. After antigen retrieval, the tissues were subjected to autofluorescence quenching and incubated in serum to block nonspecific antibody binding. The tissue sections were placed on slides and incubated with the above-listed primary antibodies at 4 °C overnight. Subsequently, the sections were incubated with a CY3-conjugated goat anti-rabbit secondary antibody (Servicebio; dilution, 1:300), and the nuclei were counterstained with DAPI (C0065, Beijing Solarbio Science & Technology Co., Ltd.). The secondary antibody used for α-SMA is Alexa Fluor 488(ab150113, Abcam; dilution, 1:400).

### Double immunofluorescence staining

Paraffin-embedded tissue sections were dewaxed and hydrated. The sections were incubated with citrate buffer, pH 6.0, retrieved for 45 min using an autoclave, and then rinsed with PBS with Tween-20, pH 7.4. The sections were further incubated overnight with primary antibodies against CD31(ab182981, Abcam; dilution, 1:1,000) and α-SMA (ab7817, Abcam; dilution, 1:1,000), and then stained with Alexa Fluor 594 (ab150080, Abcam; dilution, 1:400) and Alexa Fluor 488(ab150113, Abcam; dilution, 1:400)-conjugated secondary antibody. DAPI (C0065, Beijing Solarbio Science & Technology Co., Ltd.) was used as a nuclear counterstain.

### Immunohistochemistry

Paraffin-embedded tissue sections were dewaxed and hydrated. After antigen retrieval, endogenous peroxidase activity was blocked by incubating the tissues in 3% hydrogen peroxide, followed by blocking in rabbit serum for 30 min to inhibit nonspecific antibody binding. The tissues were placed on slides and incubated with primary antibodies overnight at 4 °C, followed by a 1-h incubation with a goat anti-rabbit secondary antibody (D3002, Long Island Antibody, China; dilution, 1:1,000). Subsequently, the slides were stained using a 3,3′-diaminobenzidine colour development kit (DA1010, Beijing Solarbio Science & Technology Co., Ltd.) at room temperature. Finally, the nuclei were counterstained with haematoxylin, and the samples were dehydrated and covered with glass coverslips.

### Real-time polymerase chain reaction (qPCR) analysis

Excised rat uterine tissues were homogenised in the presence of TRIzol reagent (Invitrogen, USA) according to the manufacturer’s instructions. After determining the purity and concentration, RNA was reverse-transcribed to single-strand cDNA. Real-time quantitative PCR analysis was performed using a SYBR Green PCR Kit (Yeasen Biotech Co., Ltd., China) in a total reaction volume of 10 µL, including 0.5 µL of cDNA, 5 µL of Master Mix, 1 µL of forward and reverse primers and 3.5 µL of distilled H2O. The 2-ΔΔCt method was used to determine relative gene expression. The following primers were used to detect mRNAs encoding proteins of interest: CD31, 5′-GGT AAT AGC CCC GGT GGA TG-3′ (forward) and 5′- TTC TTC GTG GAA GGG TCT GC-3′ (reverse); α-SMA: 5′-ACC ATC GGG AAT GAA CGC TT-3′ (forward) and 5′-CTG TCA GCA ATG CCT GGG TA-3′ (reverse); vimentin: 5′-CGA GTT CAA GAA CAC CCG CA-3′ (forward) and 5′- GCG CAC CTT GTC GAT GTA GT -3′ (reverse). As an endogenous control, the reference gene GAPDH (forward primer, 5′-AGT GCC AGC CTC GTC TCA TA-3′; reverse primer, 5′-GGT AAC CAG GCG TCC GAT AC-3′) was amplified to enable the normalisation and relative quantitative analysis of the expression of genes encoding CD31, α-SMA and vimentin. The following conditions were applied to the reactions using a thermocycler (Quantagene q225 real-time PCR system, Novogene, China): 40 cycles at 95 °C for 5 s, 60 °C for 20 s and 72 °C for 20 s. All reactions were performed at least three times.

### Western blot analysis

Total proteins were extracted from rat uterine tissues using radioimmunoprecipitation assay buffer (Beyotime, China) and phenylmethylsulphonyl fluoride (Beyotime, China). The total protein concentration in each resulting lysate was quantified using a bicinchoninic acid assay kit (Beyotime, China). Next, 40 µg of total proteins from each sample were loaded onto a 10% sodium dodecyl sulphate–polyacrylamide gel, subjected to electrophoresis to separate the proteins, and transferred to polyvinylidene fluoride membranes (Millipore, Germany). The membranes were incubated in a 5% non-fat milk solution to block nonspecific antibody binding and incubated overnight at 4 °C with primary antibodies specific for the following proteins: α-SMA (A7248, ABclonal; dilution, 1:1,000), vimentin (A19607, ABclonal; dilution, 1:1,000) CD31 (A2104, ABclonal; dilution, 1:1,000) and GAPDH (60,004–1-lg, mouse monoclonal, Proteintech; dilution, 1:4000). After three 10-min washes with Tris-buffered saline containing Tween-20, the membranes were incubated with appropriate horseradish peroxidase-conjugated secondary antibodies (ABclonal, China). Specific proteins were detected by treating the blots with enhanced chemiluminescence reagents (Biosharp, China) and exposing them to film. The protein expression levels were analysed quantitatively using AlphaEaseFC software (Alpha Innotech, USA), and the protein levels were normalised against the level of GAPDH in each sample.

### Statistical analysis

All measurements are shown as means ± standard deviations, and each set of data was derived from at least three independent experimental replications. Data from different groups were analysed using t tests and one-way ANOVAs, followed by post hoc comparisons using Dunnett’s test. SPSS 23.0 software (v.23.0; SPSS Inc., Chicago, IL, USA) was used for data processing and statistical analysis. GraphPad Prism 8.0 software (v.8.0; GraphPad Software Inc., San Diego, CA, USA) was used to create and process the histograms. A p value < 0.05 was considered to indicate a statistically significant difference.

## Results

### Successful establishment of a rat intrauterine adhesion model via the double injury method

Our experimental design included four control groups: IUA-L, Sham-L, Sham-R and the blank control group. These controls were included to demonstrate that surgical sutures alone do not cause IUA; rather, the adhesions are caused by surgical injury combined with infection. The experimental results revealed no significant difference between the four control groups (Fig. 3a, S1, S2); therefore, we selected the blank group as the control group.

**Fig. 1:**
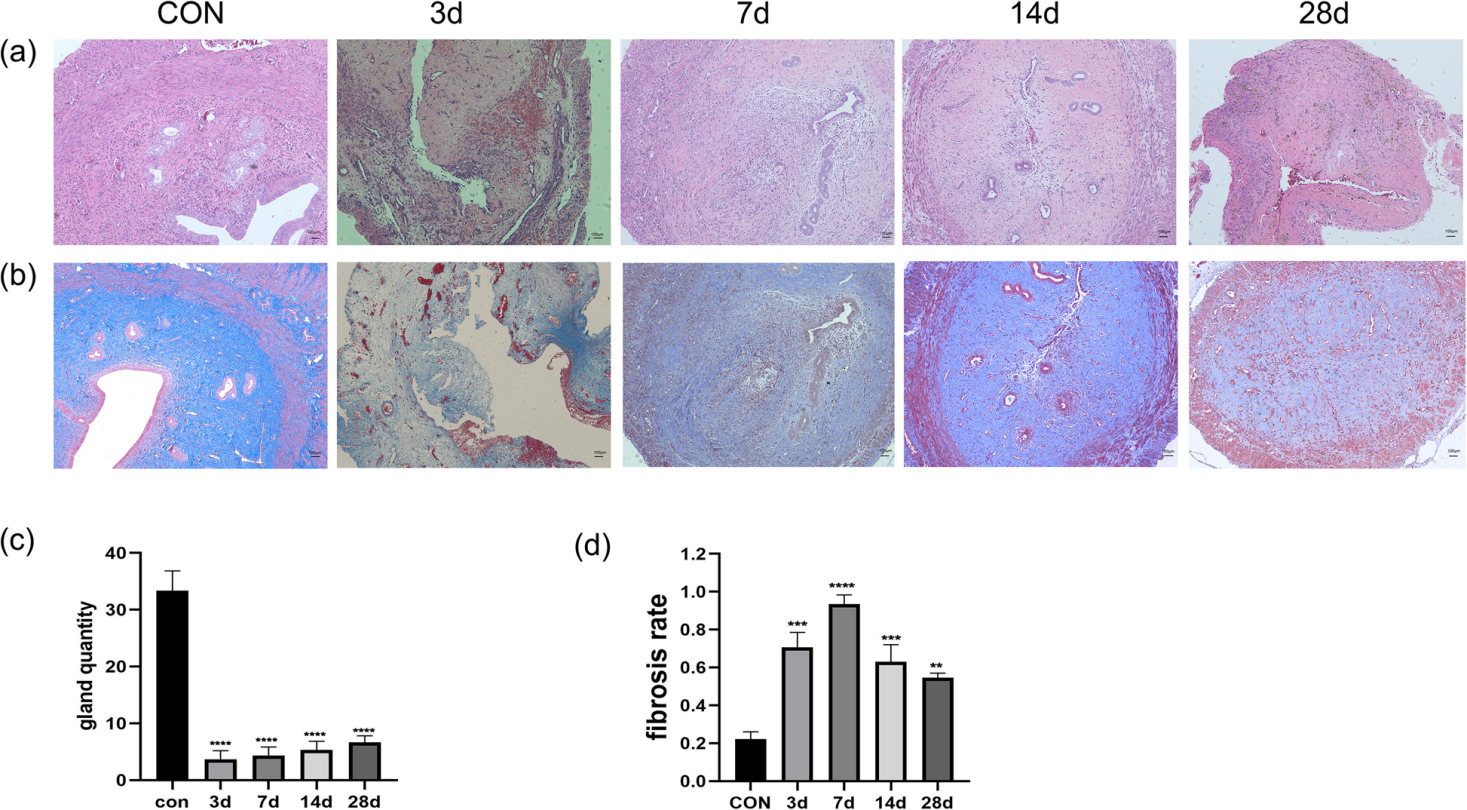
Morphological changes and fibrotic alterations in the rat endometrium after intrauterine adhesion model induction (**a**) HE staining of rat uterine tissues on days 3, 7, 14 and 28 after model induction. Scale bar = 100 µm. (**b**) Masson staining of rat uterine tissue on days 3, 7, 14 and 28 after surgery. Scale bar = 100 µm. (**c**) Changes in the number of uterine glands in rat uterine tissue after model induction. ****: *p* < 0.0001 compared with the control. (**d**) Fibrosis rate in rat uterine tissue after surgery. Fibrosis rate = blue fibrosis area/total uterine area. **: *p* < 0.01 compared with the control, ***: *p* < 0.001 compared with the control, ****: *p* < 0.0001 compared with the control.

In the rats, surgery severely damaged the endometrium and part of the myometrium, and the extent of damage was similar to that observed with clinical abortion. Therefore, our animal model is consistent with IUA due to clinical curettage. HE staining revealed that surgical damage resulted in endometrial agenesis, as demonstrated by dramatic decreases in the numbers of glands and blood vessels (Fig. 1a, c). Masson staining of the uterine tissue marked the glands and vessels in dark red and the endometrial interstitial fibres in blue (Fig. 1b). In the images, darker and wider blue-coloured areas indicate more severe fibrosis. Three days after model induction, images of the uterine cavity revealed detached tissue debris, massive fibrin exudation, inflammatory cell infiltration, a slight increase in collagen fibres, and extensive endometrial interstitial haemorrhage. After 7 days, adhesive bands were visible in the uterine cavity, which was reduced or even completely closed, and the area of fibrosis was significantly increased compared with that in the control group. After 14 days, the degree of fibrosis slightly decreased (Fig. 1d), but the uterine adhesion zone continued to increase and the uterine cavity continued to shrink. After 28 days, there was no significant improvement in the morphology of the uterine cavity compared with the previous one, and intrauterine scar formation was observed.

### Decreased levels of endothelial markers in animal models

**Fig. 2:**
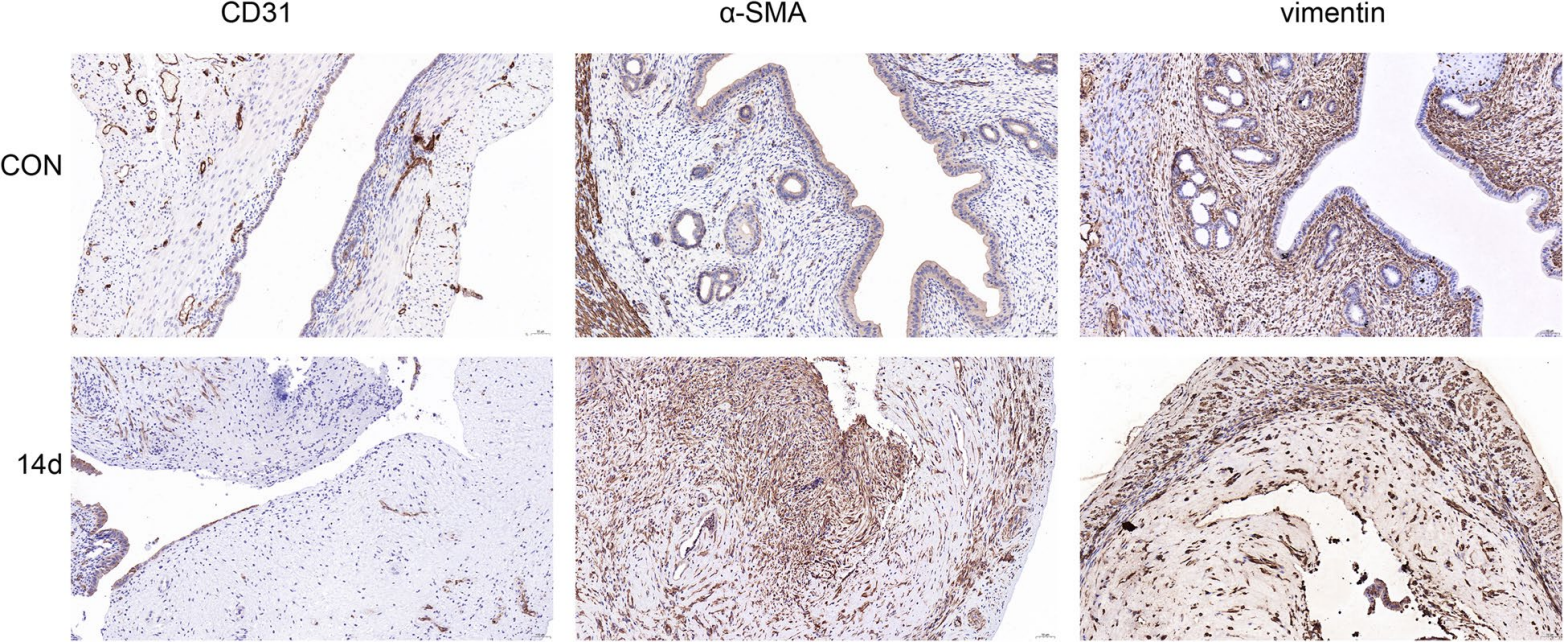
Immunohistochemical detection of EndMT-related markers. Scale bar = 50 µm

**Fig. 3:**
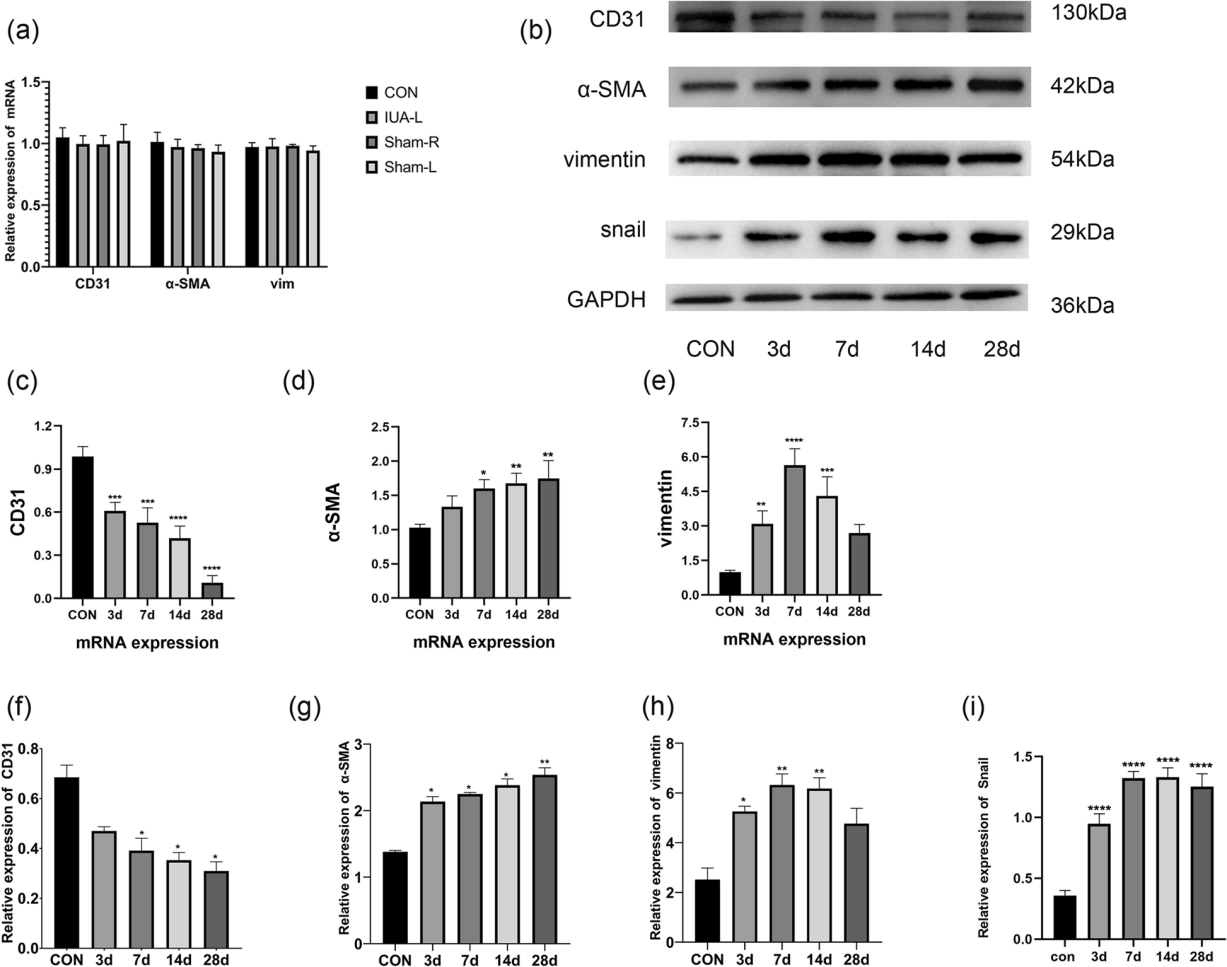
EndMT-related marker mRNA and protein levels. (**a**). Expression levels of the mRNAs encoding CD31, α-SMA and vimentin in the four control groups. There was no statistically significant difference between the controls. (**b**, **f**, **g**, **h**, i) Protein expression levels of CD31, α-SMA, vimentin, snail and GAPDH in the blank control group and 3, 7, 14 and 28 days after model induction. *: *p* < 0.05 compared with the control, **: *p* < 0.01 compared with the control. (**c**, **d**, **e**) Expression levels of mRNAs encoding CD31, α-SMA and vimentin in blank controls and 3, 7, 14 and 28 days after model induction. *: *p* < 0.05 compared with the control, **: *p* < 0.01 compared with the control, ***: *p* < 0.001 compared with the control, ****: *p* < 0.0001 compared with the control

Immunohistochemistry revealed a significantly lower level of the endothelial marker CD31 (yellowish circles) at 14 days after modelling, compared with the control group (Fig. 2). The qPCR results revealed significantly lower expression of the gene encoding CD31 at 3 days after modelling, compared with the level in the control group, and this expression continued to decrease across subsequent time points (Fig. 3c). Western blotting revealed that the trend in CD31 protein levels was consistent with the qPCR results, although the differences in this protein level between the groups were only significant beginning on day 7 after model induction (Fig. 3b, f).

### Increased levels of mesenchymal markers in animal models

**Fig. 4:**
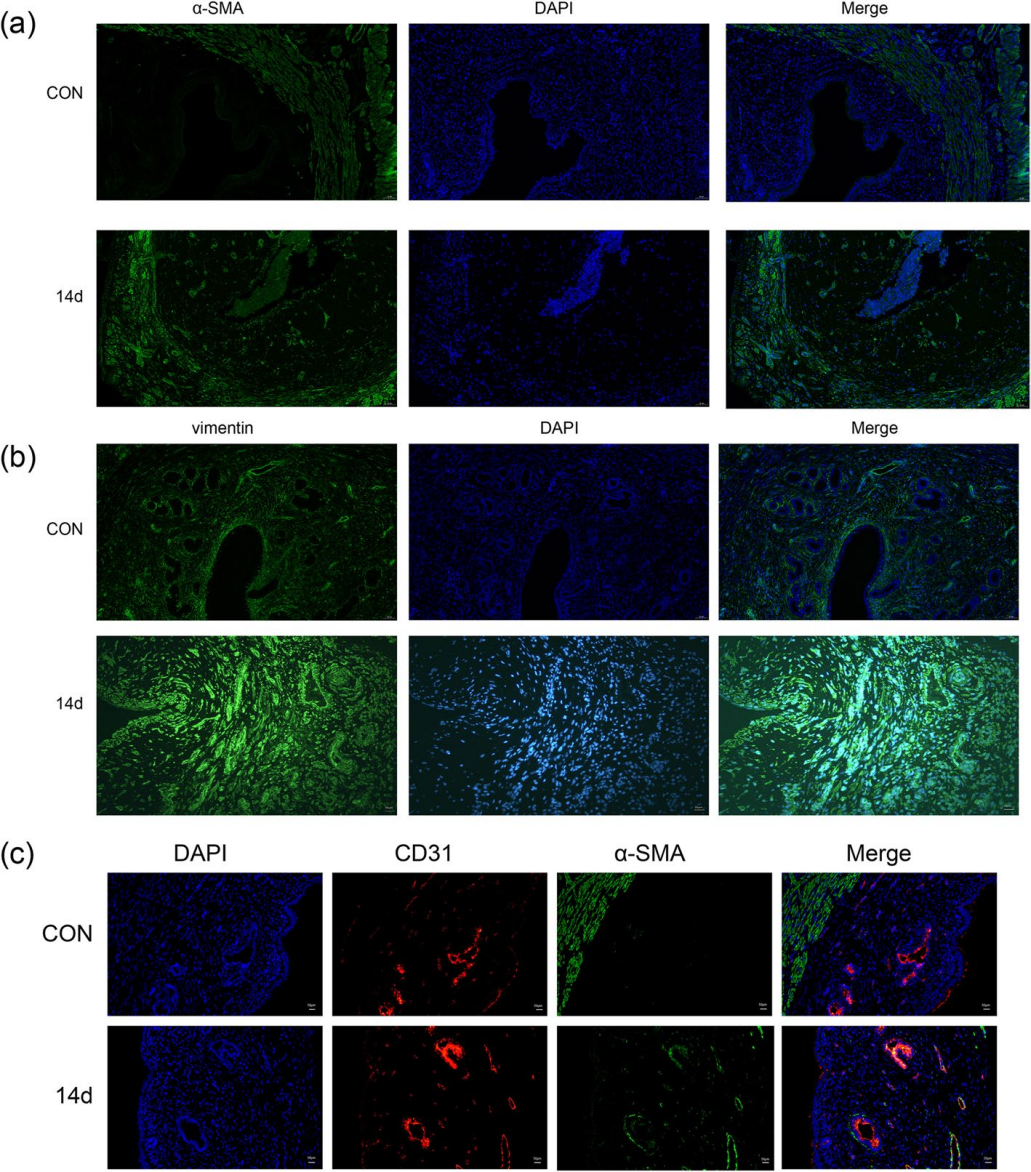
Immunofluorescence analysis of EndMT-related marker expression (**a**) Analysis of uterine tissue collected 14 days after model induction; α-SMA and nuclei (DAPI) are shown in green and blue, respectively. Scale bar = 50 µm. (**b**) Analysis of uterine tissue collected 14 days after model induction; vimentin and nuclei (DAPI) are shown in green and blue, respectively. Scale bar = 50 µm. (**c**) After modelling, EndMT-derived fibroblasts were observed in the endometrium. Images of dual-labelled tissues show α-SMA (green), CD31 (red), endothelium-derived fibroblasts (α-SMA + CD31; yellow) and nuclei (DAPI, blue). Scale bar = 50 µm.

An immunohistochemical analysis of the mesenchymal markers α-SMA and vimentin revealed significant increases in both proteins in the endometrium at 14 days after model induction, compared with the control group (Fig. 2). The results of the immunofluorescence analysis were consistent with the immunohistochemistry findings (Fig. 4a, 4b). The qPCR results revealed that compared with the control group, the level of expression of the gene encoding α-SMA began to increase after 3 days of modelling; this difference relative to the control became statistically significant on day 7 and continued to increase throughout the experimental period. In contrast, the expression of the gene encoding vimentin increased to a peak on day 7 and then decreased as the tissue repaired itself (Fig. 3d, 3e). The results of Western blotting were consistent with those of qPCR (Fig. 3b, 3g, 3h).

### Fibroblasts originate from endothelial cells in the IUA model

To demonstrate that fibroblasts originate from endothelial cells, we performed an immunofluorescence analysis to evaluate the co-localisation of CD31 andα-SMA. After 14 days, we could clearly observe CD31 and α-SMA double-positive cells in the endometrium of tissues from the IUA model, whereas no such cells were present in tissues from the control group (Fig. 4c; red = CD31, green = α-SMA, yellow = CD31 and α-SMA co-localisation). In addition, we detected changes in the expression of snail, a transcription factor of EndMT (Fig. 3b, 3i, S3). The expression of snail was significantly elevated after endometrial injury, this result again proved the presence of EndMT in the endometrial fibrosis process in IUA.

## Discussion

In this study, rats subjected to both uterine surgery and induced infection developed severe uterine fibrosis and adhesions. Subsequent immunofluorescence and immunohistochemistry analyses of relevant endothelial and mesenchymal markers revealed changes in the localisation and levels of EndMT-related molecules. Our results thus confirm the involvement of EndMT in endometrial fibrosis. We further detected changes in the expression of genes encoding EndMT-related markers and the encoded proteins via qPCR and Western blotting, respectively. Specifically, after model induction, we observed persistent decreases in both the expression of the gene encoding CD31 and the related protein, and persistent increases in both the expression of the gene encoding α-SMA and the related protein. Although vimentin expression peaked on day 7 and then declined, the levels of this mesenchymal marker remained consistently higher in the IUA model group than in the control group, further confirming the involvement of EndMT in IUA.

Although the incidence of IUA increases each year, effective treatment options to reduce the incidence of re-adhesion and promote fertility remain lacking [9]. Potentially, activated fibroblasts may be derived from resting fibroblasts in the tissue, via epithelial-to-mesenchymal transformation, or from the bone marrow [25], [35, 36]. In this study, we demonstrate for the first time that endothelial cells also promote the emergence of fibroblasts during endometrial fibrosis in uterine adhesions via EndMT. Studies have shown that inhibitors of EndMT can reduce or even reverse fibrosis by inhibiting various growth factors and regulating various transcription factors associated with the initiation or development of this differentiation process [37–40]. These inhibitors may offer new hope for the clinical treatment of IUA. Further studies are needed in this area.

One limitation of our study is the inadequate number of experimental animals. However, after pre-experimentation, we were able to control the experimental conditions carefully, ensuring that the vast majority of rats developed severe uterine adhesions postoperatively. Therefore, our results are reliable, despite the small sample size. In addition, our rats largely developed severe blockages in the uterine cavity. It is possible that our experimental findings might not be applicable to minor intrauterine adhesions, and further study is needed to address this limitation.

IUA remains a challenge and serious threat to women’s fertility and reproductive health worldwide [20]. The basal endometrial layer has a strong capacity for proliferation and repair. However, some types of intrauterine surgery, such as curettage, damage the basal endometrial and even the myometrial layers; this damage, when combined with inflammation-induced fibrous exudation, a large increase in fibroblasts, and endometrial scar repair, can seriously hinder the complete regeneration of uterine glands, blood vessels and epithelial cells, eventually leading to intrauterine adhesions. Therefore, studying the origin of the infiltrating fibroblasts and blocking the related pathways is crucial to promote regenerative endometrial repair.

Myofibroblasts usually disappear after successful tissue repair, whereas the dysregulation of normal repair processes can lead to persistent myofibroblast activation, such as chronic inflammation or mechanical stress in the tissue [25]. EndMT leads to the uncontrolled conversion of endothelial cells to mesenchymal cells and further to myofibroblasts [41, 42]. Notably, α-SMA, which is expressed in the normal myometrium but not usually in the endometrium, is usually associated with fibroblast activation. In this study, our immunohistochemistry and immunofluorescence analyses revealed abnormally high levels of α-SMA in the endometrium of rats in the IUA model group, which was suggestive of fibroblast proliferation. Vimentin, a mesenchymal marker, is normally expressed in the cytoplasm of stromal cells but is absent from epithelial cells. a typical mesenchymal feature of IUA is the distinct presence of waveform proteins in epithelial cells. Here, we found that wave proteins appeared in epithelial cells after model induction, whereas they were absent in cells from the control animals. EndMT is a transformational process: at a given time, endothelial markers and fibroblast markers can be observed simultaneously. During the transitional state in which endothelial cells are transformed into mesenchymal cells, the number of fibroblasts of endothelial origin exceeds our observation capabilities. However, our immunofluorescence experiments, in which rat uterine tissues were dual-labelled to detect co-localised CD31 and α-SMA expression, reliably demonstrated that some fibroblasts were indeed derived from endothelial cells.

## Conclusion

In conclusion, our experiments demonstrate for the first time that EndMT occurs during the progression of IUA, and enriches the underlying fibrotic mechanism. Inhibiting or even reversing this process would greatly alleviate endometrial fibrosis. Our findings provide new insights into the development of drugs for the clinical treatment of IUA.

## Data Availability

All data generated or analysed during this study are included in this published article [and its supplementary information files].

## Abbreviations

IUA: Intrauterine adhesion
EndMT: Endothelial-to-mesenchymal transition
α-SMA: Alpha-smooth muscle actin
CD31: Platelet and endothelial cell adhesion molecule 1
TGF-β: Transforming growth factor beta
EMT: Epithelial-to-mesenchymal transition
qPCR: Real-time polymerase chain reaction
IUA-R: In the IUA groups, the right uterine horn of rat was subjected to double damage.
IUA-L: In the IUA groups, the left uterine horn of rat was was not treated in any way.
Sham-R: In the sham groups, the right uterine horn of rat was subjected to sham surgery.
Sham-L: In the sham groups, the left uterine horn of rat was was not treated in any way
