# Correction to: EndMT: New fndings on the origin of myofbroblasts in endometrial fbrosis of intrauterine adhesions

**DOI:** 10.1186/s12958-022-00904-7

**Published:** 2022-02-11

**Authors:** Chengcheng Xu, Meng Bao, Xiaorong Fan, Jin Huang, Changhong Zhu, Wei Xia

**Affiliations:** Institute of Reproductive Health, Tongji Medical College, Huazhong University of Science and Technology, Wuhan, 430030 China

**Correction to: Reprod Biol Endocrinol 20, 9 (2022)**

**https://doi.org/10.1186/s12958-022-00887-5**

Following publication of the original article [1], the authors reported an error in Fig. 1. The panels “b, c and d” were misplaced in the image. The correct version of Fig. 1 is presented below.

**Fig. 1:**
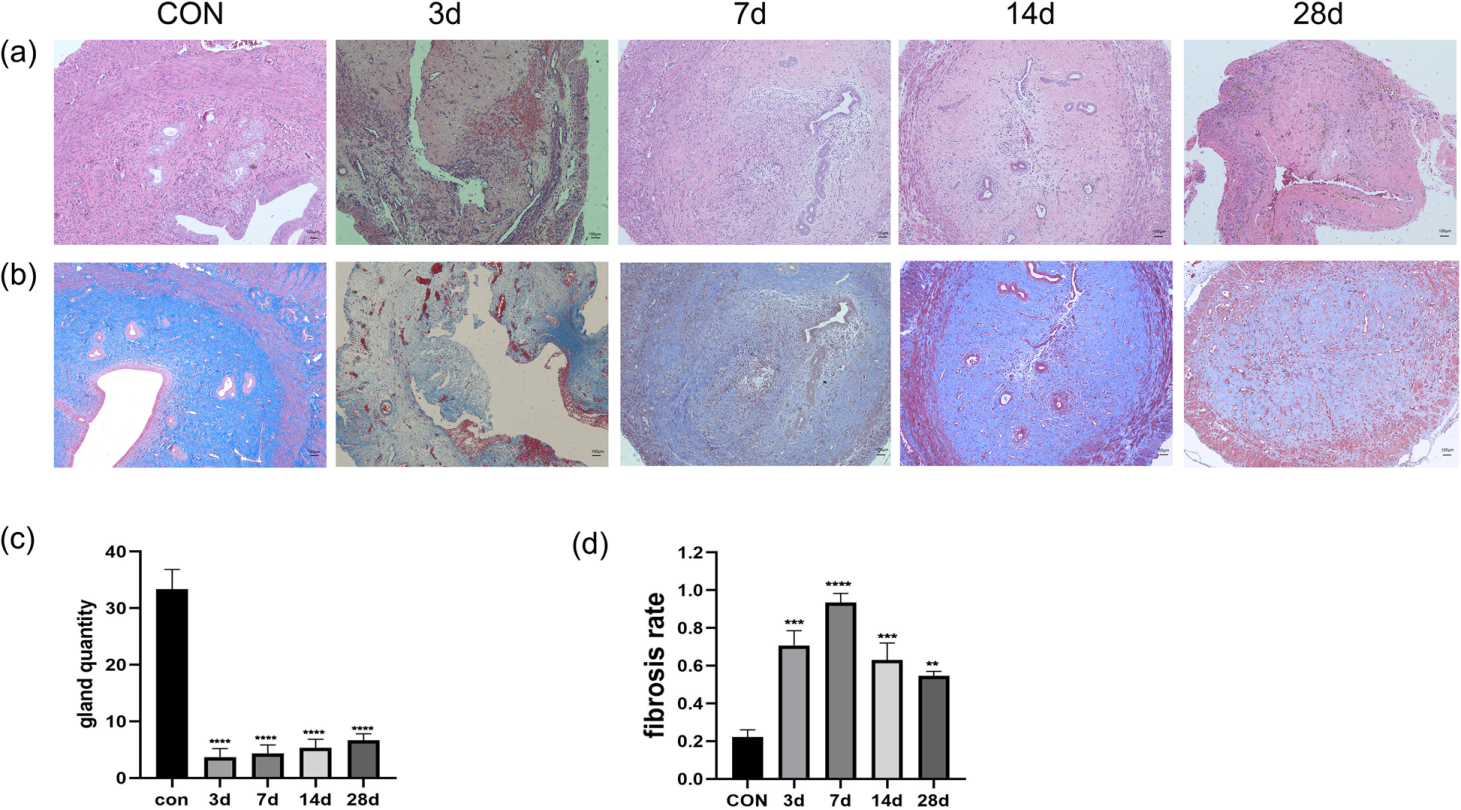
Morphological changes and fibrotic alterations in the rat endometrium after intrauterine adhesion model induction **a** HE staining of rat uterine tissues on days 3, 7, 14 and 28 after model induction. Scale bar = 100 μm. **b** Masson staining of rat uterine tissue on days 3, 7, 14 and 28 after surgery. Scale bar = 100 μm. **c** Changes in the number of uterine glands in rat uterine tissue after model induction. ****: *p* < 0.0001 compared with the control. **d** Fibrosis rate in rat uterine tissue after surgery. Fibrosis rate = blue fibrosis area/total uterine area. **: *p* < 0.01 compared with the control, ***: *p* < 0.001 compared with the control, ****: *p* < 0.0001 compared with the control

The original article [1] has been updated.
